# Distributed Drone Base Station Positioning for Emergency Cellular Networks Using Reinforcement Learning

**DOI:** 10.1007/s12559-018-9559-8

**Published:** 2018-05-22

**Authors:** Paulo V. Klaine, João P. B. Nadas, Richard D. Souza, Muhammad A. Imran

**Affiliations:** 1 School of Engineering, University of Glasgow, Glasgow, UK; 2 Federal University of Santa Catarina (UFSC), Florianópolis, Brazil

**Keywords:** Emergency communication network, Machine learning, Reinforcement learning, Unmanned aerial vehicles

## Abstract

Due to the unpredictability of natural disasters, whenever a catastrophe happens, it is vital that not only emergency rescue teams are prepared, but also that there is a functional communication network infrastructure. Hence, in order to prevent additional losses of human lives, it is crucial that network operators are able to deploy an emergency infrastructure as fast as possible. In this sense, the deployment of an intelligent, mobile, and adaptable network, through the usage of drones—unmanned aerial vehicles—is being considered as one possible alternative for emergency situations. In this paper, an intelligent solution based on reinforcement learning is proposed in order to find the best position of multiple drone small cells (DSCs) in an emergency scenario. The proposed solution’s main goal is to maximize the amount of users covered by the system, while drones are limited by both backhaul and radio access network constraints. Results show that the proposed *Q*-learning solution largely outperforms all other approaches with respect to all metrics considered. Hence, intelligent DSCs are considered a good alternative in order to enable the rapid and efficient deployment of an emergency communication network.

## Introduction

Although sporadic, natural large-scale disasters, such as earthquakes, hurricanes, and tsunamis, produce a profound impact in human society, not only in terms of the infrastructure that is destroyed, but most importantly, in terms of human lives that are lost. Whenever a disaster occurs, it is crucial that search and rescue teams are deployed in a very quick and effective manner, as the first 48 to 72 h after a disaster, also known as the *golden hours*, are the most critical [1, 2]. However, one major problem that arises during this period is the lack of communication infrastructure, as most of the existing network can be destroyed during a disaster, degrading the rescue team’s effectiveness and ability to find isolated people. Hence, in order to overcome a disaster situation, it is also vital that an emergency communication network (ECN) is deployed as fast as possible, so that communication can be restored quickly, preventing additional casualties.

In general, ECNs must be extremely adaptable, flexible, and intelligent in order to adjust themselves to the environment and situations that they can be inserted in. Hence, conventional solutions that are found today in wireless cellular networks may not be applicable, as deploying a completely new network from the very beginning can take several days. This can occur either due to the conditions in which the environment is, such as a city being completely devastated, limiting the accessibility to certain areas, and by consequence, limiting network coverage, or due to the complex process of configuring and setting up all new base station (BS) parameters. Thus, more robust solutions, involving networks that are capable of self-organization and that can be deployed quickly and effectively to the exact area where coverage is needed, should be designed. In this sense, algorithms that can adapt themselves, such as artificial intelligence and machine learning, should be deployed [1–4], to enable a fully autonomous network.

One possible solution for a rapid deployment of an ECN that can provide the desired flexibility, quickness, and intelligence is the utilization of drones with wireless capabilities [1, 2, 5–7]. These drones, also known as drone small cells (DSCs), would be equipped with a small BS and would serve as radio access points in the network. In addition, since after a disaster reaching certain locations of the affected area can be problematic, due to debris blockage or flooding, for example, positioning conventional BSs can be a rather difficult task for network operators. Thus, because of their flexibility and mobility, drones can perform a crucial role in emergency situations, by flying to the affected area and providing service exactly where it is needed.

In this paper, a positioning algorithm for DSCs in an emergency situation is proposed. The solution aims at finding the best position of multiple drones in a scenario where the old cellular network infrastructure was completely destroyed and users are in need of coverage. The DSCs are considered to have limited resources in both radio access network (RAN) and backhaul. In addition, users could have different requirements in terms of throughput and different mobility characteristics, depending if the user is from a rescue team or a regular user. Based on that, a distributed algorithm, based on reinforcement learning (RL), more specifically *Q*-learning, is proposed, so that the drones can explore the affected area and find the best possible position. This way, the main objective, to maximize total network coverage (or minimize the number of users in outage), can be achieved. The proposed solution is compared to different positioning strategies, such as deploying the drones in fixed random positions, fixed around a circle centered in the middle of the area at evenly spread angles, and deploying the drones in the locations of hot spots of the previous destroyed network, and the results show that the intelligent *Q*-learning solution outperforms all of them in all considered metrics.

### Related Work

Aerial platforms, such as drones, are expected to have an important role in the next generation of mobile networks. Because of their flexibility, adaptability, and mobility capabilities, these platforms can be deployed in a wide range of situations, ranging from providing extra coverage and capacity whenever a big event takes place, supplying the necessary communication infrastructure in case of an emergency, or bringing service in rural and isolated areas, to name a few. Because of these reasons, the deployment of drones in mobile communication networks has seen an increased attention recently [1, 3, 5–15]. In addition, the deployment of machine learning solutions in cellular networks, more specifically self-organizing cellular networks, has also seen an increase in recent years and research groups all over the world are developing intelligent solutions in order to tackle the various challenges of cellular networks [4].

Erdelj et al., in [1], present a survey of the advances in drone technology focused on wireless sensor networks and disaster management. The survey divides a disaster into three main stages and presents drone applications and challenges for each one of them. In [8], the authors show key aspects of the design and implementation of future aerial communication networks; however, instead of focusing on small drones, the authors focus on Helikite platforms.

Other works, such as [9–11], attempt to find the best position of DSCs analytically. In [9], for example, the authors attempt to find the best position for low altitude platforms (LAPs) in order to maximize their coverage range. The authors develop an analytical solution to determine the best altitude of a LAP and end up concluding that the optimum altitude is strongly dependent on the environment. Mozaffari et al., in [10], derive the optimal altitude of DSCs which gives the maximum coverage, while minimizing the transmit power. The system is investigated in two different scenarios, one considering interference between drones and another being interference-free. Results showed that, when interference is considered, there is an optimal separation distance between drones in order to maximize the network coverage. In [11], Alzenad et al. present an optimal placement algorithm for DSCs that maximize the coverage while minimizing the transmit power of the drones. In addition, the authors also decouple the problem in two, considering the placement of the drones as two separate problems in both horizontal and vertical dimensions. Results show that their system is able to save a significant amount of power, while also increasing the number of covered users.

Kalantari et al., in [3], propose to find the best position of DSCs, but instead of determining it analytically, they utilize a particle swarm optimization (PSO). Their results show that the algorithm is capable of adapting to different scenarios and that the drones were able to find by themselves the best positions in order to maximize the number of users being covered. Ahmadi et al., in [5], propose a novel mobile network architecture, considering drones as a core part of the network. Their work formulates the optimum placement of drones, while also presenting some challenges and future research directions. Also, regarding the positioning of drones, Merwaday et. al. show in [12] that, in an emergency scenario, finding the optimal position for temporary DSCs via exploiting the mobility of the drones yields improvements in network throughput and spectral efficiency.

Another work by Kalantari et al., in [13], investigates the usage of flying base stations considering different types of backhaul links. The authors introduce two different approaches, mainly a network-centric approach and a user-centric approach, and determine the best 3D position of DSCs. Their results show that the network-centric approach is able to maximize the number of covered users and that the user-centric solution maximizes user throughput. Another paper which considers backhaul limitations is the work in [11], by Alzenad et al., wherein the authors study the feasibility of a novel backhaul framework considering aerial platforms and free-space optics point-to-point links. Their results demonstrate that this type of backhaul is capable of delivering higher data rates than others, but it is also very sensitive to the environment, including clouds and fog. In [7], the authors consider the utilization of drones as a complementary approach to future terrestrial mobile networks. The authors present some design opportunities and challenges and also develop a case study on the positioning of DSCs.

Mozaffari et al., in [6], present the deployment of a drone network on top of an already existing device-to-device network. The authors evaluate the system in two different scenarios, considering static and mobile drones. The authors derive the outage and coverage probabilities for each case and show that the mobile strategy performs better than the static one in terms of coverage and energy efficiency. Azari et al., in [14], propose a framework for the analysis and optimization of air to ground systems considering altitude and cooperation diversity. The authors consider drones as relays and develop analytical solutions for the drones’ height in order to maximize its reliability and coverage range. Lastly, Shah et al., in [15], propose a new solution to the problem of user cell association considering flying BSs with backhaul constraints. The authors present a distributed solution based on a greedy search algorithm and show that the proposed approach has better results than other baseline approaches and it is less computational complex.

Regarding the application of intelligent techniques, a particular family of algorithms that has gained a lot of attention recently are the ones based on RL. Because of their capability of online learning despite the environment they are inserted in, RL algorithms can be applied in many different domains. One example of application is the one in [16], in which the authors use *Q*-learning together with deep learning to develop an algorithm that can play several Atari 2600 games, like *Pong* and *Breakout*. By taking only the raw pixels of the screen as inputs, the authors were able to show that their algorithm was capable of learning by itself how to play each game and was even able to outperform previous approaches and beat human experts in some games.

Another example is the work in [17], in which the authors propose a brain-inspired cognitive architecture for autonomous learning of knowledge representation. This architecture presents key concepts in terms of acquiring knowledge based on behavioral needs and reusing patterns to explain new situations. Results show that their implementation is able to solve simple problems, but the authors state that this approach might be better in terms of scalability of more complex tasks. In [18], the authors describe an approach to control a robot based on the actor critic algorithm. The proposed method is tested in a landmark approach, involving movable cameras, which successfully control two types of robots in performing a navigational task. Results show that the proposed solution is capable of performing autonomous navigation and highlighted the possibilities toward a more independent robot control in the future. Moreover, Zhao et al., in [19], propose a general computational model inspired by the human brain and RL concepts. The proposed algorithm is verified in a drone application, in which drones had to fly through specific paths, such as through windows and doors in order to avoid certain obstacles.

In the context of wireless networks, several intelligent solutions are being proposed. The work in [20], for example, proposes a novel cognitively inspired clustering algorithm in order to manage the energy consumption of a wireless sensor network. However, shifting the focus toward the applications of RL algorithms in cellular networks, the works by Jaber et al., in [21–23], are a good example. In these works, the authors propose a *Q*-learning solution in order to tackle the problem of user cell association considering backhaul constraints. By adjusting the offsets of small cells in order to allocate users with different requirements to the best fitting cell, based not only in RAN requirements, but also in backhaul parameters, the proposed solution is able to mitigate user dissatisfaction at a slight reduction in total perceived throughput.

Despite some works covering the deployment of drones in emergency situations [1, 8], other works covering the deployment of drones with backhaul limitations [11, 13, 15], and others considering the positioning of aerial platforms [3, 5–7, 9–11, 14], only [3] proposes an intelligent solution in order to determine the best position of DSCs. Also, as it can be seen from the reviewed literature, most studies address the drone positioning problem analytically, through the development of closed-form equations. These methods, although important, require several assumptions, such as the knowledge about how many users are in the network and their positions. In addition, most of these works also do not take into account user mobility and perform the drone placement optimization for a specific, static scenario. Hence, these types of solutions might not be suitable for real situations, in which the environment is constantly changing, users can move at different speeds, and even network parameters, such as cell load and backhaul conditions, can change as well.

In addition, as previously mentioned, the only work that proposes an intelligent solution to the problem of drone positioning optimization is the work of Kalantari et al., in [3]. However, the proposed work utilizes a PSO algorithm, which can be viewed as a branch of genetic algorithms or heuristic methods (in constrast to genetic algorithms, PSO does not perform selection in between generations) [24, 25]. Although able to solve the proposed problems in a simulated environment, solutions such as GA, heuristics, and PSO, due to their inherit nature of having to search for the best possible solution among a family of available ones, are not suitable for applications that require continuous interaction between the system and its environment. This occurs because any change in the initial original set of solutions would require the whole computation to be performed again. For instance, PSO is not able to perform an online optimization of the problem.

As the authors show in [3], the approach is tested in two fixed scenarios, without considering user mobility. Because PSO performs an offline computation, this solution is also not capable of adapting itself to real-time changes in the network. For example, if mobility was taken into account, the proposed PSO algorithm would have to run again, every time a user would move, in order to determine the best new solution for this new network configuration, resulting in an impractical system. Additionally, due to the vast search space that the PSO solution has to evaluate, a centralized unit would be required in order to perform all the required computations and determine the best configuration. Again, in real systems this is not practical, as this would result in an increase in communication signaling between the centralized unit and the drones, as well as the need of synchronization. Lastly, due to the heuristic nature of PSO, this approach would also not be scalable as well as computationally efficient due to the vast search space that it must compute in order to find the best possible configuration. In a real environment, for example, in which network conditions and user positions change frequently, PSO would not be able to cope with these changes, becoming an impractical solution in real scenarios.

Based on the issues mentioned above, it is clear that the development of a novel solution that is capable of adapting itself online and that is also able to analyze the environment and determine the best possible actions to be taken is needed. Based on that, RL algorithms are a suitable approach since, independently of the environment they are inserted in, they can explore the possibilities and determine the best actions to be taken.

### Objectives and Contributions

The main objective of this paper is to provide a distributed and intelligent solution to the problem of positioning multiple DSCs in order to maximize the number of covered users in an emergency situation. As previously mentioned, this problem is of high importance in emergency situations; since the fastest communication network can be established, more human lives can be saved. This optimization problem also poses a difficult challenge, due to the varying conditions of the environment, such as users moving with different speeds, users having different requirements, and the DSCs being limited in both RAN and backhaul resources. In addition, as the deployment of conventional BSs might not be possible, either due to the infrastructure being destroyed or parts of a city not being accessible, the deployment of drones is considered to be a good solution in case of emergency situations.

In order to provide a solution to the problem of drone 3D placement, the development and evaluation of an adaptable and flexible solution based on RL using *Q*-learning are proposed. The developed algorithm is shown to be robust enough in order to adapt itself to different network conditions, such as the position of other DSCs, interference between DSCs, user movements, and requirements. To the best of the author’s knowledge, there are no other works that consider the optimization of drone positioning using RL in an emergency communication scenario, while also considering user mobility, user requirements, and network constraints.

The main novelty of this work can be described as the development of an intelligent solution based on RL, in order to tackle the problem of user coverage in an emergency situation. As discussed before, most of the current state-of-the-art solutions do not provide the needed flexibility or adaptability in order to cope with a changing environment; hence, the development of an intelligent solution that is capable of providing coverage exactly where and when needed is essential. The proposed solution utilizes a Q-learning algorithm in every DSC and each DSC will attempt to find the best position in the environment so that the global reward, given by the total number of users covered, is maximized. Results show that the proposed solution achieves better results than fixing the drones either in random positions, in a circular manner, or in the location of the previous hot spots.

The remainder of this paper is organized as follows. Table 1 contains a list of symbols used throughout the paper, “Method” describes the system model, “Proposed solution” presents the proposed solution and the metrics used to measure the performance of the system, while “Results” discusses numerical results. Lastly, “Conclusions” provides a summary of the key findings of the paper and some suggestions for future work.

**Table 1:** List of symbols

Symbol	Definition
Scenario	
$\mathbb {B}$	Set of all base stations
*L*	Side of the considered area
*N*_B_	Number of base stations
*N*_u_	Number of users
$\mathbb {U}$	Set of all users
ITU-R	
*α*	Ratio of buildup to land area
*β*	Building density
*γ*	Scale parameter for building height distribution
*W*	Width of buildings
*S*	Separation between buildings
Link	
*B*	Bandwidth
*c*	Speed of light
*C*_h_	Antenna height correction factor
*d*_m/d_	Distance between user and macro cell/drone
EIRP	Equivalent isotropically radiated power
*f*_c_	Carrier frequency
*h*_B_	Base station height
*h*_d_	Drone height
*h*_u_	Height of user device
*N*	Additive white Gaussian noise power
PL_m/d_	Path loss between user and macro cell/drone
RSRP	Reference signal received power
$\phantom {0}\rho $	Drone coverage radius
SINR	Signal to interference plus noise ratio
*T*	Throughput
*𝜃*	Drone antenna major lobe angle
*ξ*	Additional path loss
Algorithm	
*a*	Action
*ε*	Chance of choosing a random action
*ϕ*	Discount factor
*λ*	Learning rate
MAX_it_	Max iterations per episode
MAX_it,r_	Max iterations with same reward
MIN_it_	Min iterations per episode
*Q*	Action-value function
*r*	Reward (total number of users allocated)
*s*	Agent state
*t*	Time instant
Performance metrics	
*D*_*τ*_	Average throughput dissatisfaction
*D*_u_	Percentage of users in outage
*N*_o_	Number of users in outage
Ψ	Set of unsatisfied users in terms of throughput
*τ*	User required throughput

## Methods

### System Model

The problem of maximizing coverage in an emergency situation scenario, via the deployment of a temporary network is considered. This network is composed of a truck BS, along with movable DSCs. The objective of the proposed system is to find the best possible positions of DSCs, given that users with different requirements and mobility characteristics are distributed in the scenario and that both the truck BS and the DSCs have limited resources in terms of RAN and backhaul.

#### Urban Model

The International Telecommunication Union (ITU-R) defines in [26] three parameters to characterize any urban environment, which are defined as:

- $\alpha $, the ratio of buildup land area to the total land area;
- $\beta $, the average number of buildings per square kilometer;
- $\gamma $, scale parameter for the heights of the buildings.

**Fig. 1:**
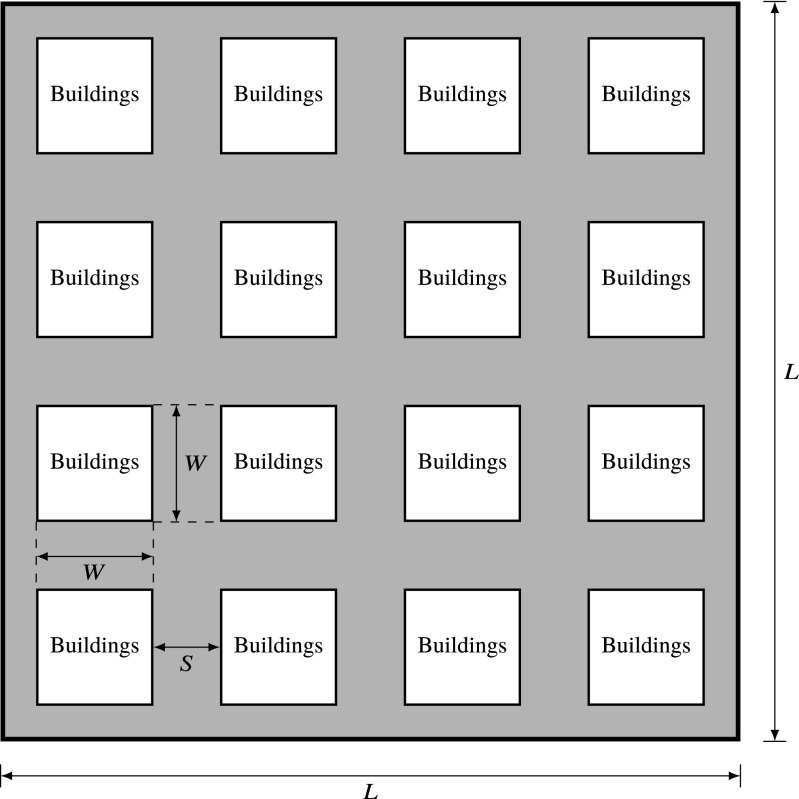
Manhattan grid urban layout

Following [27], the urban scenario in this paper is modeled considering these parameters, and a building disposition following a Manhattan grid layout, as in Fig. 1, in which squares of a given width (*W* ) are separated by a distance (*S*) [28]. The height of the squares is obtained following a Rayleigh distribution with scale parameter $\gamma $, whereas *W* and *S* are assumed equal across all buildings and are determined via [27]

1 $$ W = 1000\cdot \left( \frac{\alpha}{\beta}\right)^{\frac{1}{2}} $$

and

2 $$ S = \frac{1000}{\sqrt{\beta}}-W. $$

### Users

The scenario considers $N_{\text {u}}$ users spread across an *L* by *L* square area. A portion of the users is assumed to be concentrated near hot spots randomly distributed around the area, while the rest of the users is uniformly random distributed.

In addition, two types of users are considered: either users that belong to an emergency team, or regular users. Regardless of their type, the most important requisite for all of them is to be connected (to have coverage). Aside from that, rescue team users can have different throughput requirements, depending on their needs, while normal users are all assumed to have low throughput requirements. Furthermore, different mobility levels are assumed between users. Regular users, for example, are considered to have low mobility, whereas rescue team users are assumed to have high mobility.

### Temporary Network

The scenario considered in this paper assumes that there was a fully functional network in a certain location, but due to a natural disaster, it was completely destroyed. In this particular case, however, it is assumed that either part of the original backhaul of the previous network was still accessible or that a backhaul link could be deployed after the emergency happened and could be used by network operators in order to establish a connection to their servers. In other situations, in which the original backhaul links of the previous network are also destroyed, a solution can be developed first, to deal with the backhaul connection issue, and then the proposed solution, involving the deployment of drones, can be installed.

#### Truck Base Station

In order for operators to access the original backhaul of the network, a fully equipped truck with a macro BS powered either by fuel or solar energy is considered, such as in [29]. This truck could be placed in a position similar to where the original BS was positioned and, by connecting to the available backhaul link, it can enable the connection between the truck BS and the network operator.

The path loss from the macro BS to the users follows the Okumura-Hata model [30]

3 $$\begin{array}{@{}rcl@{}} \text{PL}_{\text{m}} = 69.55 + 26.16\cdot\log_{10}\left( f_{\text{c}}\right)-13.82\cdot\log_{10}\left( h_{\text{B}}\right)-C_{\text{H}}+\\+[44.9-6.55\cdot\log_{10}\left( h_{\text{B}}\right)]\log_{10}\left( d_{\text{m}}\right), \end{array} $$

where $f_{\text {c}}$ is the carrier frequency, $h_{\text {B}}$ is the height of the macro BS, and $C_{\text {H}}$ is the antenna height correction factor, which is given by

4 $$ C_{\text{H}} = 0.8+\left( \log_{10}\left( f_{\text{c}}\right)-0.7\right)\cdot h_{\text{u}}-1.56\cdot \log_{10}\left( f_{c}\right), $$

where $h_{\text {u}}$ is the height of users’ mobile devices.

#### Drone Small Cells

Additionally to the truck BS, it is also considered that DSCs are deployed in the network in order to provide the additional needed coverage. Each drone is considered to have a dedicated out of band backhaul link, composed of a microwave link, which is able to contact the truck BS in order to connect to the network operator. The traffic from the drones are routed to the truck BS and then to the network operator, as the truck BS is the only one with a direct backhaul connection to the operator. Also, it is assumed that the drones use a dedicated spectrum slice of their band to perform this connection to the macro BS. Furthermore, since the drones are flying at relatively high altitudes and the antenna from the macro cell is positioned higher than nearby buildings, it is assumed that the link between drones and macro BS has very large capacity, similarly to what is considered in [12]. Moreover, as recently shown in [31], the noise and LOS characteristics of the channel affect the link between ground BS and drones far less than when considering the communication between the ground BS and a ground user, further supporting this assumption.

**Fig. 2:**
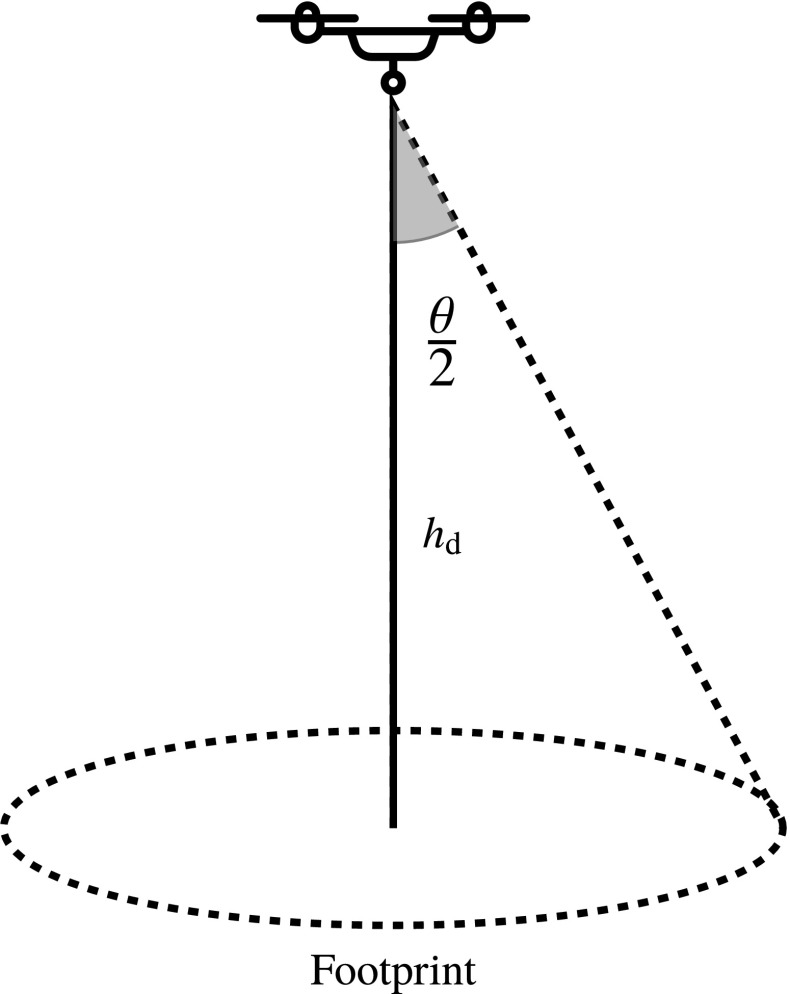
DSC flying at a height, *h*_d_, and with an antenna with aperture angle of *𝜃*

It is also considered that the antenna of each DSC has strong directivity, such that its major lobe^1^ (defined as the region in which the antenna gain is the highest) has an aperture angle of $\theta $, as in Fig. 2. Therefore, the signal irradiated from its antenna is considered highly attenuated outside its coverage radius $\rho $ defined by

5 $$ \rho = h_{\text{d}} \cdot \tan\left( \frac{\theta}{2}\right), $$

where $h_{\text {d}}$ is the drone flight altitude.

**Fig. 3:**
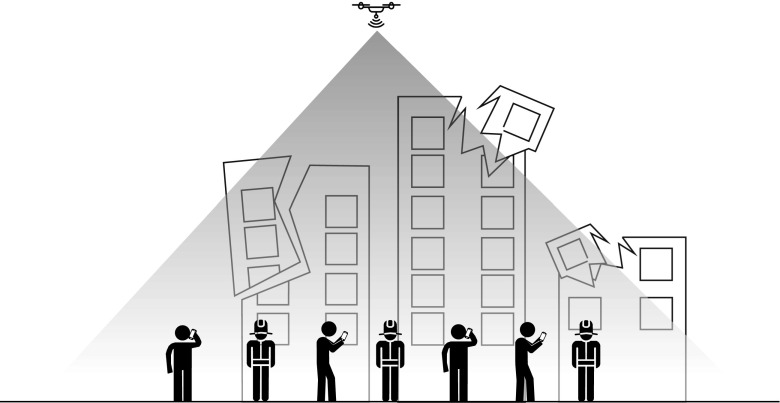
Considered scenario. A DSC providing coverage to a certain amount of users, both regular and rescue team users, in an emergency situation

The path loss between DSCs and users (PL_d_) follows the model presented by Al-Hourani et al., in [27], wherein $\text {PL}_{\text {d}}$ is split between a free-space path loss and an additional loss, which depends on whether there is line of sight (LOS) between the drone and the user or not [10]

6 $$ \text{PL}_{\text{d}} = 20\log_{10}\left( \frac{4\pi f_{\text{c}} d_{\text{d}}}{c}\right)+\xi, $$

where $d_{\text {d}}$ is the distance between drones and users, *c* is the speed of light, and $\xi $ is the additional loss which assumes different values for LOS or non-line-of-sight (NLOS) links. Figure 3 presents the scenario considered in this paper, with a DSC covering regular users and rescue team in a post-disaster urban scenario.

### User Allocation

Considering the set of all users $\mathbb {U} = {1,2,\ldots ,N_{\text {u}}}$ and the set of all BSs $\mathbb {B} = {1,2,\ldots ,N_{\text {b}}}$, with $N_{\text {b}}$ as the total number of BSs, the received signal power, in dB, for user $i\in \mathbb {U}$, from BS $j\in \mathbb {B}$, is denoted by $\text {RSRP}_{i,j}$ and can be computed as

7 $$ \text{RSRP}_{\text{i,j}} = \left\{\begin{array}{ll} \text{EIRP}_{\text{m}} - \text{PL}_{\text{m}}, &\text{if} \ j \ \text{is a macro BS}, \\ \text{EIRP}_{\text{d}} - \text{PL}_{\text{d}}, &\text{if} \ j\ \text{is a DSC}, \end{array}\right. $$

where $\text {EIRP}$ stands for equivalent isotropically radiated power and represents the transmitted power combined with its antenna gain, in dB. According to that, $\text {EIRP}_{\text {m}}$ and $\text {EIRP}_{\text {d}}$ represent the total transmit power from the macro BS and the DSCs, respectively. $\text {PL}_{\text {m}}$ represents the path loss between the macro BS and users, calculated according to (3), and $\text {PL}_{\text {d}}$ is the path loss between drones and users as given in (6).

Next, the signal to interference plus noise ratio (SINR), for a BS/user pair, $\text {SINR}_{i,j}$, is given by

8 $$ \text{SINR}_{i,j} = \frac{\text{RSRP}_{i,j}}{N+\sum \limits_{k = 1, k \ne j}^{N_{\text{b}}} \text{RSRP}_{i,k}}\quad, $$

where *N* is the additive White gaussian noise (AWGN) power and the $\text {RSRP}$s are expressed in linear form.

The throughput *T* for a user *i* allocated to BS *j*, in bits per second, is determined following Shannon’s channel capacity formula [33]

9 $$ T = B\cdot\log_{2}(1+\text{SINR}_{i,j}), $$

where *B* is the bandwidth in Hz.

As in [21], the amount of throughput that the user consumes from the backhaul is considered to be 30% higher than its actual throughput, because of overhead signals. In addition, if the sum of the backhaul throughput of any BS exceeds its capacity, i.e., if the backhaul is overloaded, the throughput of all users connected to that cell is reduced equally by taking the amount of overloaded capacity and dividing it by the total number of users connected to that cell. This guarantees that the total throughput of the cell does not exceed its capacity, while also penalizing cells having its backhaul overloaded.

Lastly, users are then allocated to the best BS according to their SINR. If the SINR of a user is above a certain threshold, and the BS has enough space in its RAN, then a user is allocated to that BS in that time slot. However, if that BS has no resource blocks available or the user SINR is too low (below the connection threshold), the next BSs are tried, in order of highest SINR. After all BSs are tried, if a user is still unable to be associated with a BS, the user is considered to be out of coverage (in outage) for that time slot.

## Proposed Solution

The goal of this work is to propose and evaluate an intelligent algorithm, based on RL, to find the best positions of multiple DSCs, which maximize the number of served users in an urban area where a disaster has occurred. RL is a type of machine learning technique based on a goal-seeking approach [34]. In contrast to supervised learning, for example, in which the system learns by analyzing a labeled data set, or unsupervised learning, in which given unlabeled data, the system tries to correctly infer the output. In RL, a system must explore the environment in order to discover which actions are the best to take at specific states [34, 35].

### Positioning Algorithm

The proposed algorithm is based on *Q*-learning, which is an off-policy, dynamic programming method. In *Q*-learning, agents are able to learn, without having a model of the environment, their optimal behavior by experiencing the consequences of their actions [36].

Each agent in the *Q*-learning algorithm has an action-value matrix, *Q* matrix, which represents the value of being in a specific state $s_{t}$, while taking an action $a_{t}$, at time *t*. By trying different actions in different states (exploring), but also by picking the best possible action at other states (exploiting), *Q*-learning is shown to converge for any type of policy being followed [34].

In the proposed solution, a distributed approach is assumed. Each DSC is considered an agent of the *Q*-learning solution, and the disaster scenario (composed of the buildings, user distribution and movement, and macro cell location) is the unknown environment that the agents are inserted in. The states of the DSCs are defined as their three-dimensional position in the environment, and each drone can take any of seven possible actions, namely move up, down, left, right, forward, backward, or not move at all. In addition, the drones follow an $\epsilon $-greedy policy [34] to choose their actions, with a decaying $\epsilon $, depending on the number of iterations.

Since the main target of the proposed solution is to maximize the amount of covered users, the reward experienced by each drone is the total of users allocated by the system. The total number of users allocated was chosen as a reward metric, instead of considering the number of users allocated by each drone, so that drones would have a better incentive to allocate more users instead of each drone being greedy and trying to maximize their own reward. If the individual number of users allocated per DSC was used as reward, this could result in a drone allocating users from other drones, so that its reward would be maximized at the expense of the reward of the others, leading to a sub-optimal (or local) optimization. Thus, a reward that represented a global metric, in this case the total number of users allocated, was chosen. A summary of the *Q*-learning parameters is provided next and Algorithm 1 summarizes the proposed solution.

#### **Agents**

Agents correspond to the DSCs. Each agent has a different *Q*-matrix, which describes the value of a drone taking an action for every given state.

#### **States**

A state *s* is the three-dimensional position of a DSC in space. In addition, for the considered model, the space is divided into a grid, such that the set of possible states a drone can be in is finite.

#### **Actions**

Each drone can take seven possible actions. These actions are moving one step in any direction (up, down, left, right, forward, backward), or to stay still. Furthermore, if an action would take the DSC out of the grid, it has the same effect as staying still.

#### **Reward**

Since the goal is to maximize the number of served users, the reward, *r*, is given by the total number of allocated users. For that to be possible, the DSCs and the macro cell are assumed to share the information of the number of allocated users with each other, via the connection with the macro BS. More formally, the reward of the system can be defined as follows:

10 $$ r = \sum\limits_{j = 1}^{N_{\text{b}}} U_{j}, $$

where $U_{j}$ is the number of users allocated to BS *j*.

#### **Update Strategy**

Each DSC updates its *Q* matrix according to

11 $$ Q(s_{t},a_{t}) \leftarrow Q(s_{t},a_{t})+ \lambda[r_{t + 1}+\phi\max\limits_{a} Q(s_{t + 1},a)-Q(s_{t},a_{t})], $$

where $Q(s_{t},a_{t})$ is the current action-value function, $\lambda $ is the learning rate, $r_{t + 1}$ is the expected reward at the next time step, $\phi $ is the discount factor, and $\max \limits _{a} Q(s_{t + 1},a)$ is an estimate of the optimal future action-value function at the next time step. The DSCs also keep track of the state which yielded the best reward.

#### **Initialization**

At the start, all the DSCs are positioned at random locations and with their *Q* matrices set to zero in all entries.

#### **Stopping Criteria**

The stopping criteria are based on three conditions. The drones have moved for a maximum number of iterations Max_it_, the value of the reward has not improved in a certain number of iterations $\text {Max}_{\text {it,r}}$, or the drone has used all its resource blocks and has explored for a minimum number of iterations $\text {Min}_{\text {it}}$. When one of them is met, the DSC moves to the state which yielded the best reward and stops until the next episode.

#### **Episode**

Whenever there is a notion of time steps in RL algorithms, the agent-environment interaction can be broken into subsequences. These subsequences are called episodes and are composed of repeated interactions between the agent and the environment, until a terminal state, or a stopping criteria, has been met [34]. In the context of the proposed problem, an episode can be defined as a snapshot of the environment, or in this case, the emergency communication network. In each episode, the DSCs take actions based on their current state and evaluate their reward. This process is repeated for a certain number of iterations until one of the three stopping criteria is met.

In addition, during each episode of the network, the users are considered to be static, so that the drones can evaluate and determine the best actions for that snapshot of the network. After all drones have moved and found the best possible positions for that episode, the current episode ends, users eventually move according to their mobility levels, and a new episode begins. Furthermore, since there is a high correlation between episodes, whenever a new episode begins, the DSCs start at the previous position of the last episode. Also, the DSCs keep their *Q* matrices between episodes, in order to explore the previous knowledge gathered from previous episodes of the network.

In a real scenario, however, as there is no notion of episodes, the DSCs could perform the position optimization every certain time intervals, for example, whenever the global reward of the system is below a certain treshold. By analyzing the network at certain time slots, and moving according to the user positions of that time slot, the DSCs would still be able to find on the fly an optimal solution. In addition, this interval (the frequency that the algorithm is run in each DSC) could also be tuned in order to have a higher or lower resolution, at the trade-off of energy and complexity at each DSC as well as based on the assumed user mobility model. 
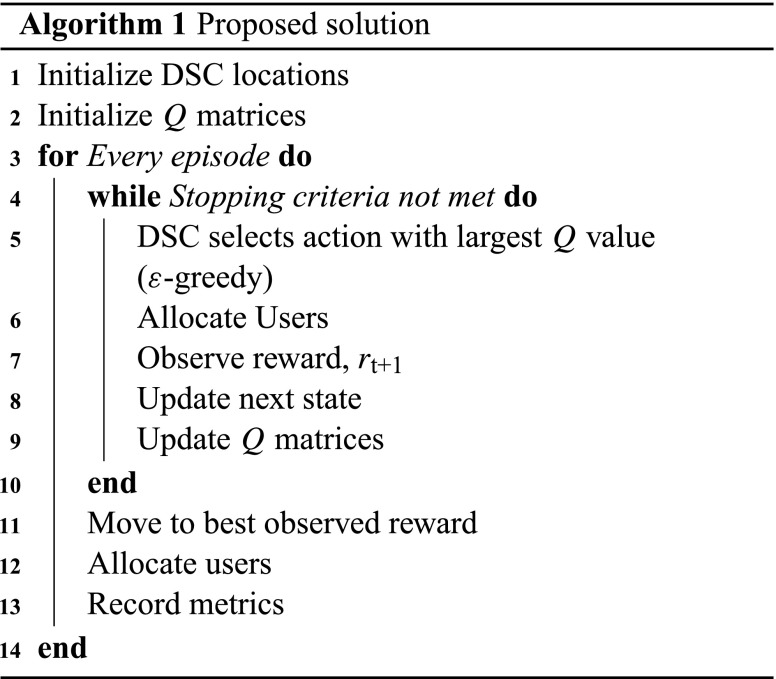


### Metrics

In order to evaluate the proposed strategy, the metrics considered are the percentage of users in outage $D_{\text {u}}$ and the average user throughput dissatisfaction $D_{\tau }$ per total number of users. The percentage of users in outage is given by

12 $$ D_{\text{u}} = 100\cdot \frac{N_{\text{o}}}{N_{\text{u}}}, $$

where $N_{\text {o}}$ is the total number of users in outage and is defined as

13 $$ N_{\text{o}} = N_{\text{u}} - \sum\limits_{j = 1}^{N_{\text{b}}} U_{j}. $$

By its turn, $D_{\tau }$ is computed considering all the users which have a throughput below his/her requirement, $\tau $, such that

14 $$ D_{\tau} = \frac{1}{N_{\textit{u}}}\sum\limits_{y \in {\Psi}} \frac{\tau_{y}-T_{y}}{\tau_{y}}, $$

where $T_{y}$ and $\tau _{y}$ are the allocated and required throughput of user *y*, respectively, and ${\Psi }$ is the set of users with $T_{y} < \tau _{y}$. In addition, if a user has not been allocated to any cell, it is considered dissatisfied and his/her perceived throughput is assumed to be $T_{y} = 0$.

## Results

### Simulation Scenario

In order to showcase the effectiveness of the proposed solution, a simulation scenario in MATLAB has been built. It is considered that a network operator had a previously fully functional network in the area, composed of a macro and several small cells. However, a disaster happened and the previous network was fully destroyed, with only parts of its original backhaul connection available, so the operator has to choose other means in order to restore connectivity as fast as possible.

**Fig. 4:**
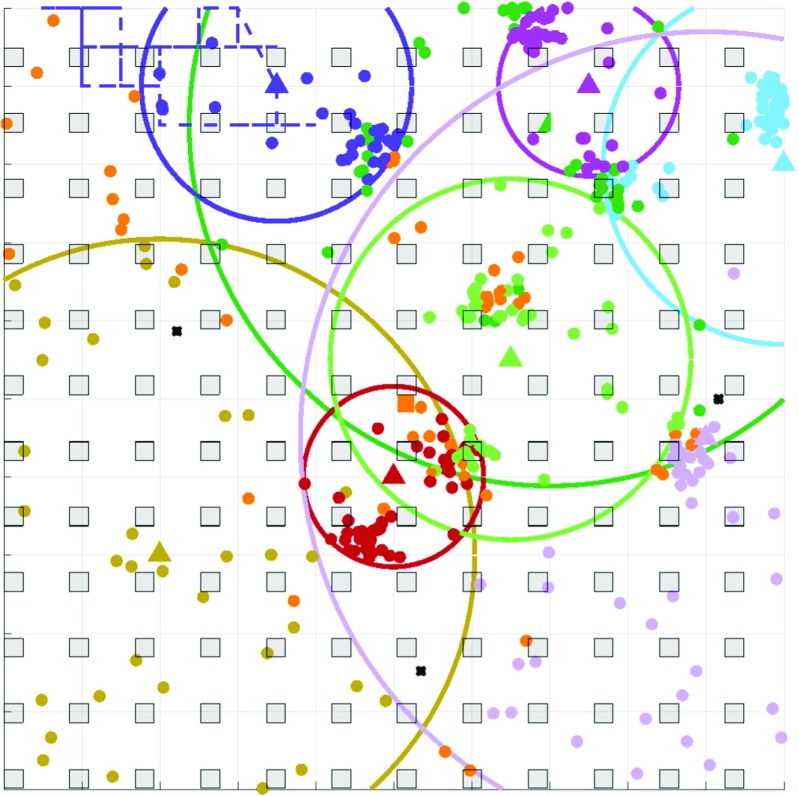
Upper view of the simulation scenario. The macro cell, in orange, is positioned near the center of the area, while the drones are shown as colored triangles. The DSC coverage radius is represented as the colored circles and users served by the BSs (either truck BS or DSCs) are displayed with different colors, while users in outage are represented as black X’s. The trajectory of one drone is plotted (dashed)

**Fig. 5:**
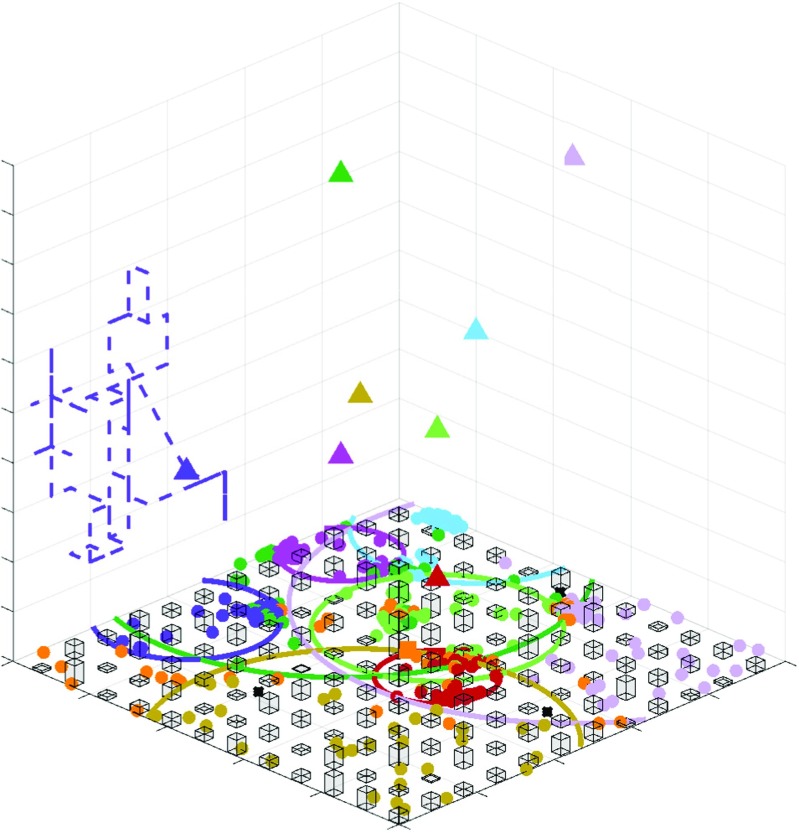
Isometric view of the simulation scenario. DSCs adjust their 3D position in order to maximize the amount of users covered. As it can be seen, different DSCs prefer different heights, in order to minimize interference between DSCs while also maximizing their coverage. The trajectory of one drone is plotted (dashed)

For this scenario, it is considered that the operator chose to deploy a macro cell in a temporary fixed infrastructure, as for instance in a truck, in a position similar to the original one. Since it might be difficult for the operator to deploy the macro BS in its original position, either due to debris or blockages, in the simulations, the truck containing the macro BS is positioned at its initial position added to an offset depending on a random distribution. In addition, drones are also deployed in the network to perform the role of the previous small cells. Regarding the drones positioning, several approaches are tested, such as fixing the drones in random positions, fixing the drones in a circular manner around the macro cell, fixing the drones in the previous location of the small cells, and, lastly, deploying movable and intelligent drones using the proposed *Q*-learning solution. The proposed solution is compared to those baselines as there are no other methods, to the best of the author’s knowledge, that perform the optimization of drone positioning in an emergency scenario, considering both RAN and backhaul constraints. We remark that the only other similar approach, the PSO-based scheme in [3], would not be a feasible and appropriate solution to the proposed scenario, as it is not able to cope with the changes in the environment, nor scalable enough to be an adequate solution to a real situation. A summary of the simulation parameters is shown in Table 2 and Figs. 4 and 5 show the simulation scenario setup in MATLAB.

**Table 2:** Simulation parameters

Parameters	Value
Ratio of buildup to total land area, *α*	0.3 [26]
Average number of buildings, *β*	500 buildings/km^2^ [26]
Scale parameter for building heights, *γ*	15 m [26]
*ξ* LOS	1 dB [27]
*ξ* NLOS	20 dB [27]
Side of the square area, *L*	1 km
Drone *X*-axis step	50 m
Drone *Y* -axis step	50 m
Drone *Z*-axis step	100 m
Minimum drone height	200 m
Maximum drone height	1000 m
Low mobility users *X*-axis step	3 m
Low mobility users *Y* -axis step	3 m
Low mobility users *Z*-axis step	0 m
High mobility users *X*-axis step	10 m
High mobility users *Y* -axis step	10 m
High mobility users *Z*-axis step	0 m
Number of users, *N*_u_	768 [21, 22]
User height, *h*_u_	1.5 m
Ratio of rescue team users	20%
Number of hot spots	16
Number of DSCs	16
Ratio of users in near hot spots	2/3 [21, 22]
Macro BS EIRP	0 dBW [21, 22]
Macro BS height, *h*_B_	20 m
DSC EIRP	− 3 dBW [31]
DSC antenna directivity angle, *𝜃*	60^∘^ [32]
RBs in macro cell	50 [21, 22]
RBs in DSCs	50 [21, 22]
Macro cell backhaul capacity	100 Gbps [21, 22]
Microwave backhaul capacity per drone	37.5 Mbps/drone [21, 22]
Bandwidth of one RB	180 kHz [21, 22]
Carrier frequency, *f*_c_	1 GHz
High SINR requirement	5 dB
Low SINR requirement	0 dB
Total number of episodes	100
Number of independent runs	100
Max iterations per episode, Max_it_	1000
Max iterations, same reward, Max_it,r_	100
Min iterations per episode, Min_it,r_	200
Learning rate (*λ*)	0.9
Discount factor (*ϕ*)	0.9

#### The Environment

The simulated scenario consists of an urban area of 1 km^2^, following the model and parameters described in [27]. In this area, 768 users are scattered, some belonging to rescue teams and others consisting of regular users. Furthermore, different users have different characteristics in terms of both mobility and throughput requirements. As the throughput can be calculated in terms of the perceived SINR, as in Eq. 8, user requirements are modeled as SINR requirements instead of throughput requirements. Table 3 presents the different types of users, requirements, and mobility levels.

**Table 3:** User characteristics

	User types	
	Rescue team	Regular
Mobility	High	Low
SINR	High/low	Low

#### User Distribution

A third of the users is randomly distributed in the entire 1-km^2^ area, whereas the other two thirds are randomly assigned to hot spots. In order to generate the hot spots, a defined number of hot spots are artificially created, and every hot spot has the same amount of users.

#### Frequency Bands and Antenna

Regarding frequency bands, it is assumed that both drones and macro cell would share the same frequency band, meaning that drones and macro cell would interfere with each other and a frequency reuse factor of 1 is considered. However, in order to mitigate the interference between drones, it is also considered that each drone has a single antenna with an elevation and horizontal plane apertures of $\theta = 60^{\circ }$, which is a good approximation of commercially available antennas [32]. This means that each drone has a fixed radius of coverage, varying with its altitude, and that users out of that radius of coverage would perceive a very low signal coming from that drone.

#### RAN and Backhaul

In addition, the scenario also assumes that both macro and DSCs are limited in both RAN and backhaul resources. Both macro cell and DSCs are assumed to share a 10-MHz bandwidth, which correspond to a capacity of 50 resource blocks (RBs), according to long-term evolution (LTE) parameters. Moreover, it is considered that the macro cell has an ideal backhaul [21, 22] and that the DSCs have a microwave link that connects to the macro cell.

#### Simulation

In order to implement the *Q*-learning solution to the problem, first, a discretization of the proposed environment is performed. In terms of user mobility, it is considered that users could move in steps of 1 m in any direction. It is also considered that the user mobile phone distance to the ground is of 1.5 m height. Regarding the DSC movements, the aerial space is discretized in steps of 50 m in the horizontal plane (*X* and *Y* dimensions) and in steps of 100 meters in the vertical domain (*Z* dimension). Drones could then either move in this space in all three dimensions or stay still.

The simulation is ran for 100 independent runs, each with a total of 100 episodes (snapshots of the network). First, however, before the drones start moving and determining their best positions, an initialization process is performed. Before the simulation begins, user positions, requirements, and mobility levels are generated. Then, a certain number of hot spots are generated with an equal amount of users per hot spot and a fixed number of drones is also positioned in the system, according to the scenario being evaluated. In addition, the backhaul of the drones is also initialized according to the microwave backhaul parameters and each drone is assumed to have a random initial backhaul load, of up to $10\%$ of its initial capacity.

For the fixed position scenarios, the drones remain in their locations for all episodes, while users move around according to their mobility behaviors. On the other hand, for the *Q*-learning approach, the DSCs try to find the best possible position for every episode. For this to be possible, however, each episode is also divided into iterations. For every iteration, the drones move around the environment looking for the best positions in the system, and during this process, the users are considered to be static. Every drone performs a certain amount of iterations, according to the stopping criteria of the algorithm and determines the best position for that episode. After all drones stop moving, the episode is finished, all metrics are recorded, and user positions are updated, so that the mobility of users can be taken into account between episodes. Lastly, whenever a new episode begins, the DSCs start from the previous learned position and with the previous computed *Q* matrices, in order to explore the correlation between different snapshots of the network. This process is then repeated and the results are averaged out between different runs of the algorithm.

### Numerical Results

**Fig. 6:**
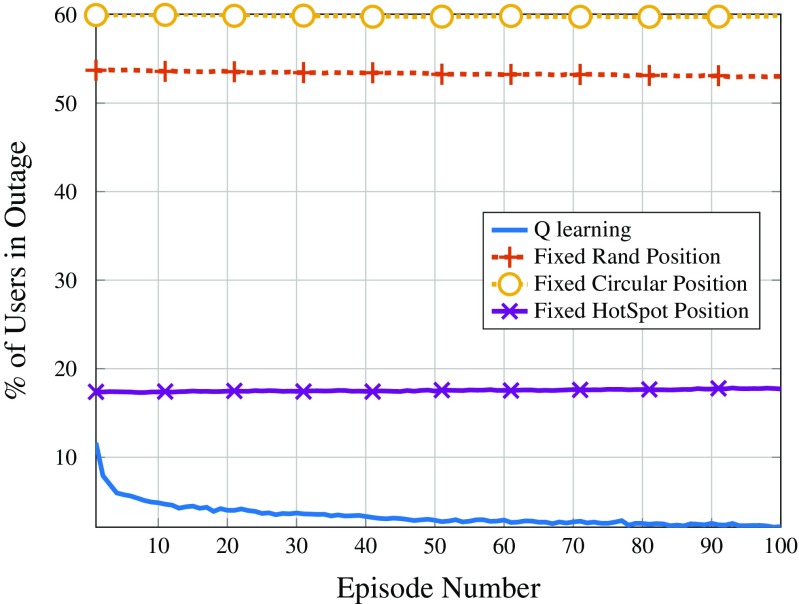
Average number of users in outage per episode

Figure 6 shows the average number of users in outage per episode for each of the considered strategies. As it can be observed, the *Q*-learning approach yields the best results, resulting in around 2% of users in outage after 100 episodes and down to less than 5% after only 10 episodes. In addition, it can also be seen that both the random fixed positioning strategy as well as the circular positioning strategy yield very poor performance in terms of covered users, having more than 50% of users in outage at any given episode. Additionally, as the metrics are computed at the end of the episodes (after the DSCs have performed their movements), it is natural that the *Q*-learning and random position curves start at different values, as in the former case, the DSCs move, while in the latter they do not. Keeping the drones fixed at the location of the hot spots also results in a poor performance, albeit better than the random and circular strategies.

This result emphasizes the importance of having a movable solution, because as users move through the network, the proposed solution is able to detect and track user movement, learning the best positions to be in for every episode. In addition, the proposed *Q*-learning solution also shows that it is able to provide coverage and service whenever and wherever it is needed.

**Fig. 7:**
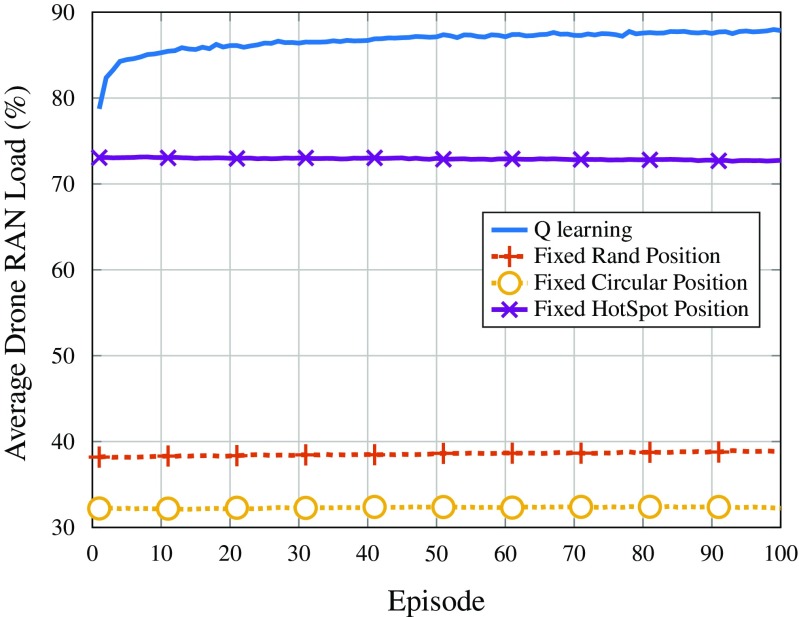
Average DSC RAN load per episode

**Fig. 8:**
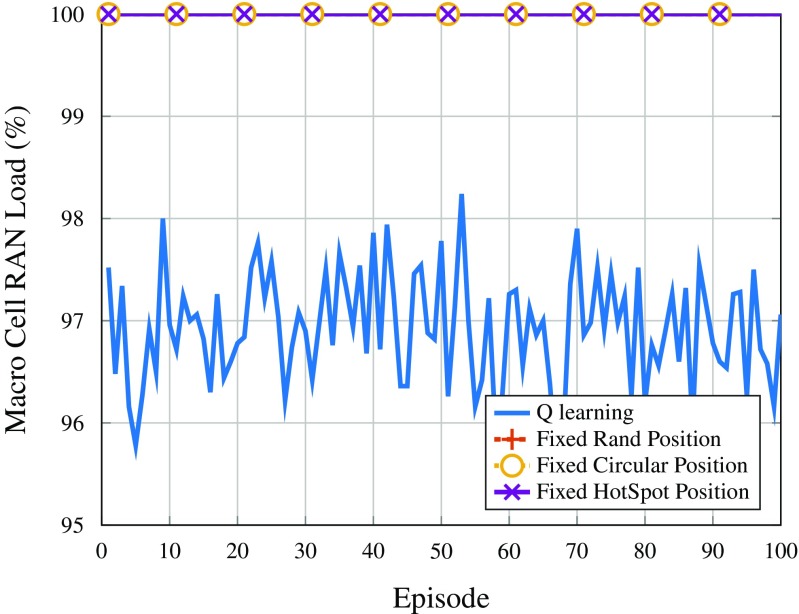
Average macro cell RAN load per episode

Figures 7 and 8 show the RAN load of the drones (averaged over all drones) and the macro cell, respectively, per episode. Regarding the *Q*-learning strategy, the RAN load of the drones increases from below 80% up to almost 90%, as can be seen in Fig. 7. This indicates that the stopping criteria being reached are not the one related to having full capacity, but rather the one in which the reward does not improve after a certain number of iterations. Moreover, this happens mainly because there is more RAN resources than the minimum required to serve all users in the network, such that the reward does not vary for longer than $\text {Max}_{\text {it,r}}$ iterations. In addition, looking at Fig. 8, it is possible to observe that the load on the macro cell almost does not vary as the DSCs find better positions in terms of reward (served users overall), indicating that the drones moving according to the *Q*-learning policy are not allocating users which were served by the macro cell and leaving others in outage, but instead they are finding users moving in a way such that users in outage can now be served.

This behavior of following users and allocating users that would be in outage, otherwise, can also be deducted from Figs. 6 and 7, by comparing the trend of the curves for users in outage and drone load. Note that both curves have inverted trends, indicating that the DSCs are using their spare capacity to provide coverage to a wider number of users. On the other hand, also regarding Figs. 7 and 8, it can be seen that the other positioning strategies have almost no variation in the curves of drone RAN load, since they do not adapt to the mobility patterns of users. Furthermore, because the RAN load on the DSCs is relatively low, the macro cell is always operating at full RAN capacity in these cases.

**Fig. 9:**
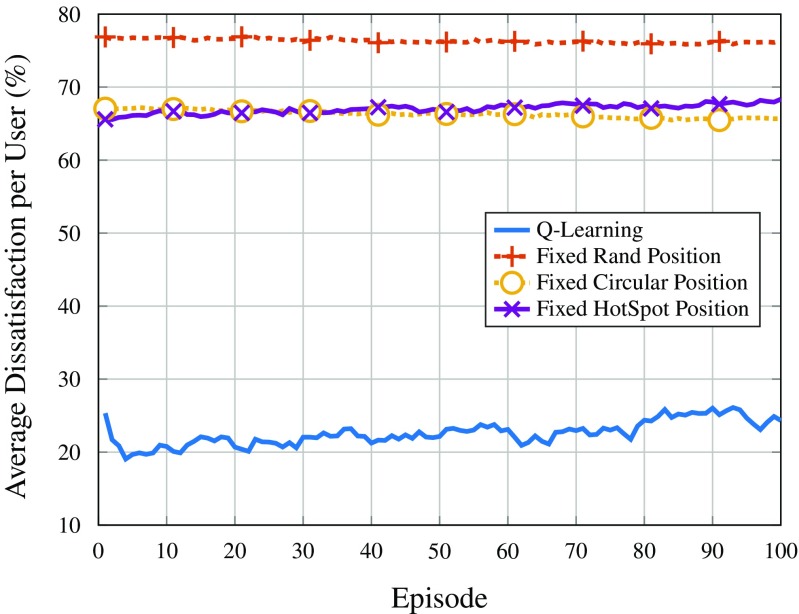
Average dissatisfaction of users with low throughput requirement

**Fig. 10:**
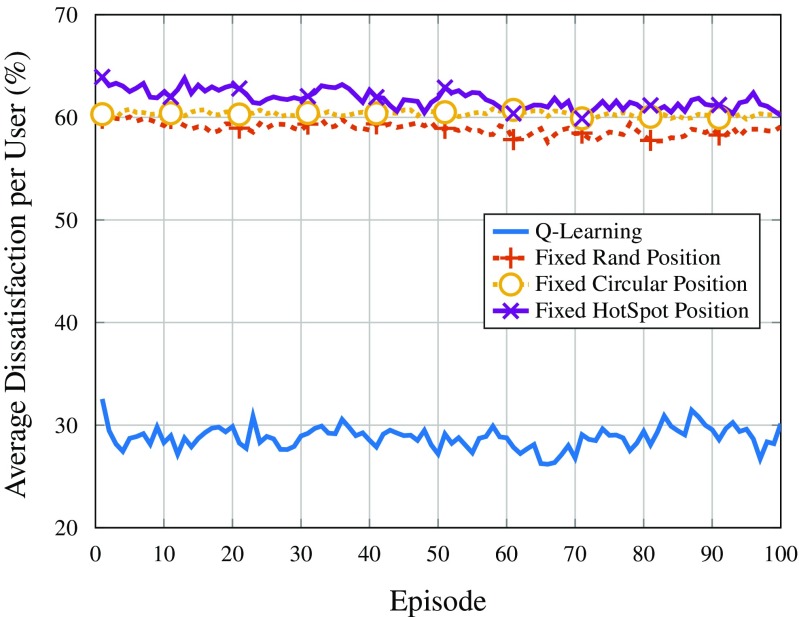
Average dissatisfaction of users with high throughput requirement

Figures 9 and 10 show the average dissatisfaction per user in terms of throughput for users with low and high requirement, respectively. As it can be seen, the *Q*-learning solution, despite not being explicitly programmed to mitigate user dissatisfaction, is the best performing strategy by a large margin. This is mainly due to the fact that users out of coverage are considered 100% dissatisfied. The performance regarding throughput satisfaction could even be improved if the reward considered this metric; however, since this is not the main goal of the proposed solution (nor the main requirement for the type of application), the *Q*-learning approach does not optimize user satisfaction.

**Fig. 11:**
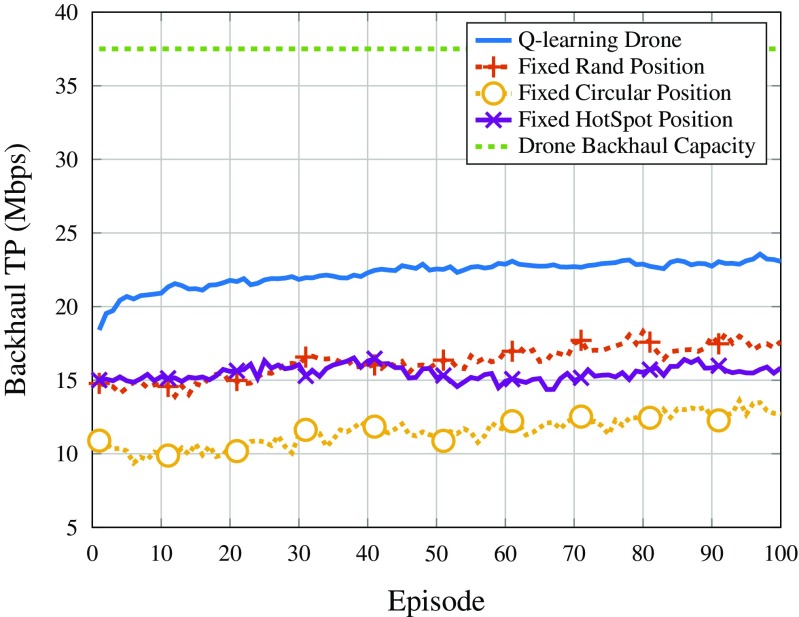
Average backhaul throughput for the drones per episode

Figure 11 shows the average backhaul throughput for the DSCs. As it can be seen, there is not a large variation of the parameters in different episodes but the important thing to note is that the backhaul capacity of the DSCs is not being exceeded, showing that the throughput bottleneck is the SINR. In addition, when comparing to the maximum possible backhaul capacity of each drone, it can be seen that the *Q*-learning strategy is the one that best utilizes the backhaul resources of the system, while the other strategies do not use the backhaul resources very well, leaving more capacity unused.

**Fig. 12:**
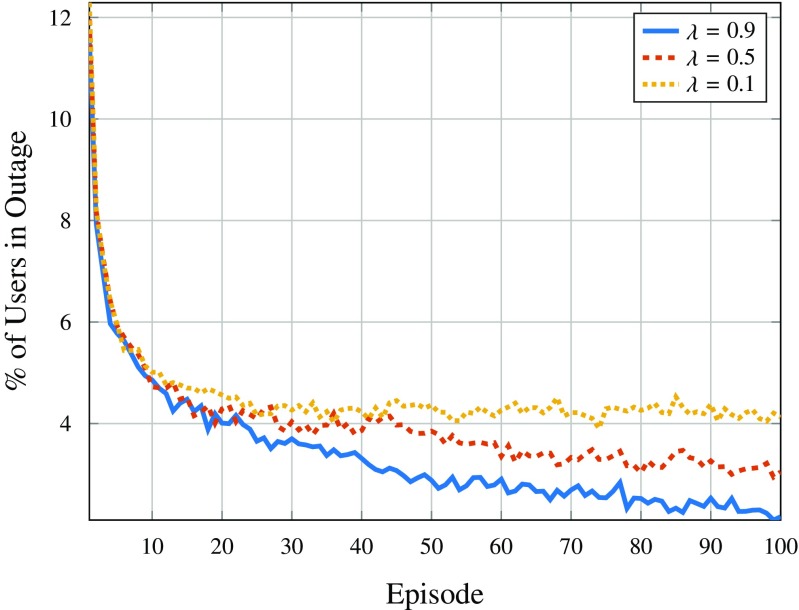
Users in outage per episode considering different learning rates for the *Q*-learning positioning strategy

The impact of different learning rates, $\lambda $, was also investigated and their influence is illustrated in Fig. 12. As expected, the learning rate exerts some influence in the convergence rate of the algorithm, as higher $\lambda $ leads to better results, as can be observed. For instance, considering $\lambda = 0.1$ results in 4% of the users being in outage after 100 episodes, whereas increasing the value of $\lambda $ gradually improves performance. This difference can be explained due to the fact that the drones learn less from the environment when smaller $\lambda $ are considered and thus are less able to adapt to the changes in the environment, such as user mobility. Hence, a value of $\lambda = 0.9$ was chosen.

## Conclusion

In order to provide service whenever large-scale natural disasters happen, it is crucial that network operators have adaptable and intelligent solutions at hand. With that in mind, new solutions have to be created, as conventional approaches and regular BSs might not be suitable or be fast enough in order to provide service in such an emergency. Hence, one possible enabler for ECNs is the deployment of intelligent drone BSs, as they can provide coverage whenever and wherever needed, due to their mobile characteristics.

In this paper, a RL approach to determine the best position of multiple DSCs is proposed. The algorithm is based on a distributed implementation of *Q*-learning. Results show that the solution outperforms other fixed methods in terms of all considered metrics. These results showcase the importance that movable BSs can have in future cellular networks, as they can learn the best positions to be in dynamic environments.

Since this is a relatively new area, the 3D placement optimization problem of DSCs in an emergency situation can have several possible extensions. One extension of this work can be the consideration of different ML techniques in order to position the drones, such as SARSA or SARSA(*λ*). In addition, RL can even be combined with other ML techniques, such as supervised learning, in what it is known as value function approximation, in order to approximate the value of every state-action pair. By doing this, the algorithm would generalize and estimate the value of every state-action pair based on features of the system, being able to determine the best actions for unseen states and also avoiding the environment discretization.

Another interesting possible future work is to consider additional parameters for the users, such as latency and resiliency and perform an optimization not only based on the connectivity of each user, but also with respect to their satisfaction levels regarding each parameter. Lastly, another future work idea can be to include additional constraints in the DSCs, such as the flight time and the total energy consumption from the DSC’s movements. This would enable the algorithm to find a trade-off between moving and coverage, in order to maximize both the amount of users and the flight time of each DSC.

## Footnotes

1 In practical antennas [32], there is one major lobe, which concentrates the majority of its power and can be defined using two angles named elevation and horizontal angles. It is assumed in this work that both have the same value, $\theta $.
